# Multiparametric Outcome Assessment After Transcatheter Aortic Valve Implantation—A Systematic Review

**DOI:** 10.3390/jcm14051426

**Published:** 2025-02-20

**Authors:** Natalia Świątoniowska-Lonc, Filip Klausa, Krzysztof Ściborski, Agnieszka Wysokińska-Kordybach, Waldemar Banasiak, Adrian Doroszko

**Affiliations:** 1 Department of Cardiology, Centre for Heart Diseases, 4th Military Hospital, 50-981 Wroclaw, Poland; k.sciborski@op.pl (K.Ś.); aga.wys@tlen.pl (A.W.-K.); banasiak@4wsk.pl (W.B.); adrian.doroszko@gmail.com (A.D.); 2 Department of Cardiac Surgery, Centre for Heart Diseases, 4th Military Hospital, 50-981 Wroclaw, Poland; filip.klausa@gmail.com; 3 Clinical Department of Cardiology, Faculty of Medicine, Wroclaw University of Science and Technology, 50-981 Wroclaw, Poland

**Keywords:** aortic stenosis, outcome, transcatheter aortic valve implantation

## Abstract

**Backround/Objectives**: Aortic stenosis (AS) is the most commonly acquired valvular disorder. Patient risk stratification and the development of an accurate and reliable tool are crucial in identifying suitable candidates for TAVI. The present review summarized the current state of knowledge on the influence of selected factors on the outcomes and course of patients with AS undergoing transcatheter aortic valve implantation (TAVI). **Methods**: The inclusion criteria for the present systematic review were as follows: (1) studies indexed in the medical databases PubMed, MEDLINE, EMBASE, CINAHL, Web of Science, and Scopus; (2) full-text articles available in English; (3) papers published between 2013 and 2023; and (4) addressing the topic of assessing the impact of factors on the outcomes of patients with aortic stenosis undergoing TAVI. This review used PRISMA 2020 reporting guidelines for systematic reviews and meta-analyses. **Results**: One hundred and thirty-two studies were eligible for this review. The available studies showed an association of psychosocial and socioeconomic factors, valve parameters, comorbidities, clinical factors, treatment-related factors, biomarkers, and treatment methods with the outcomes of patients with AS undergoing TAVI. **Conclusions**: Given the conflicting results obtained regarding the impact of right ventricular dysfunction, paravalvular leaks, and treatment method on the mortality of patients undergoing aortic valve implantation, further research in these areas is needed. In view of the researchers’ differing views on some of the factors affecting patient outcomes after TAVI, further analysis is needed to develop a new tool for assessing predictive outcomes in AS patients. This study is registered at PROSPERO (CRD42024612752).

## 1. Introduction

The latest guidelines from the European Society of Cardiology (ESC) [1] recognize TAVI as a proven alternative to surgery in patients with unacceptably high risks or contraindications to traditional surgical aortic valve replacement (SAVR). Within the past several years, TAVI has been performed much more frequently in both high-, moderate-, and low-risk patients in highly developed countries and has become a common alternative to surgical treatment [2]. Despite the widespread use of this treatment modality for patients with aortic stenosis (AS), clinicians continue to use some tools to assess the perioperative and distant risks for classic aortic valve replacement. EuroSCORE II and the Society of Thoracic Surgeons (the STS) score are the most used to predict operative mortality in cardiac surgery [3,4]; however, these regression models are of limited use in patients with an excessively high risk for cardiac procedures—a cohort that could particularly benefit from TAVI. Therefore, more attention should be paid, at least to clinical variables and anatomic conditions not included in the described predictive scales, especially for very high-risk patients undergoing TAVI [5]. As a result, patients referred for TAVI may be in a different risk group than those referred for SAVR [3]. The literature has identified some factors that may influence the outcomes of patients with aortic valve stenosis [4,6,7,8,9,10,11,12,13,14,15,16,17,18,19,20,21,22,23,24,25,26,27,28]; however, researchers consider these variables separately, which greatly limits inferences about the actual independent determinants of complications following the TAVI procedure. Therefore, patient risk stratification and the development of an accurate and reliable tool are crucial in identifying suitable candidates for TAVI.

This review aims at summarizing the current state of knowledge on the influence of numerous factors—including sociodemographic, psychosocial, and treatment-related—on the outcomes and the clinical course of procedure in patients with AS undergoing TAVI.

## 2. Methods

### 2.1. Registration, Data Availability, and Approval

This study was registered in the PROSPERO database (CRD42024612752). We did not obtain approval from the Bioethics Committee because our study used only publicly available, de-identified data. This review used the PRISMA 2020 reporting guidelines for systematic reviews and meta-analyses [29].

### 2.2. Eligibility Criteria and Search

In this review, the Patient (AS), Intervention (transcatheter aortic valve implantation), Control (not applicable), and Outcomes (quality of life, hospitalizations, mortality, readmissions) (PICO) strategy was used to develop the guiding question and research. Keywords were established according to Medical Subject Headings (MeSH) [30], and a search of medical databases was performed using the terms “stenosis” and “outcome”. The inclusion criteria were as follows: (1) studies indexed in the medical databases PubMed, MEDLINE, EMBASE, CINAHL, Web of Science, and Scopus; (2) full-text articles available in English; (3) papers published between 2013 and 2023; and (4) addressing the topic of assessing the impact of factors on the outcomes of patients with AS undergoing TAVI. Editorial letters, commentaries, study protocols, conference abstracts, review articles, and duplicates were excluded from this review.

### 2.3. Study Selection and Assessment of Risk of Bias

Selected articles were independently reviewed and considered eligible if two reviewers (N.Ś.-L. and F.K.) independently decided that they met the inclusion criteria described earlier. Disagreements were resolved by consensus or consultation with a third reviewer (A.D.) and discussion. In the first stage, all records were identified by searching electronic databases. In the next stage, two researchers (N.Ś.-L. and F.K.) independently checked titles and abstracts to identify potentially eligible studies and remove duplicates. In the third stage, potentially eligible studies were selected for a full-text review.

**Figure 1:**
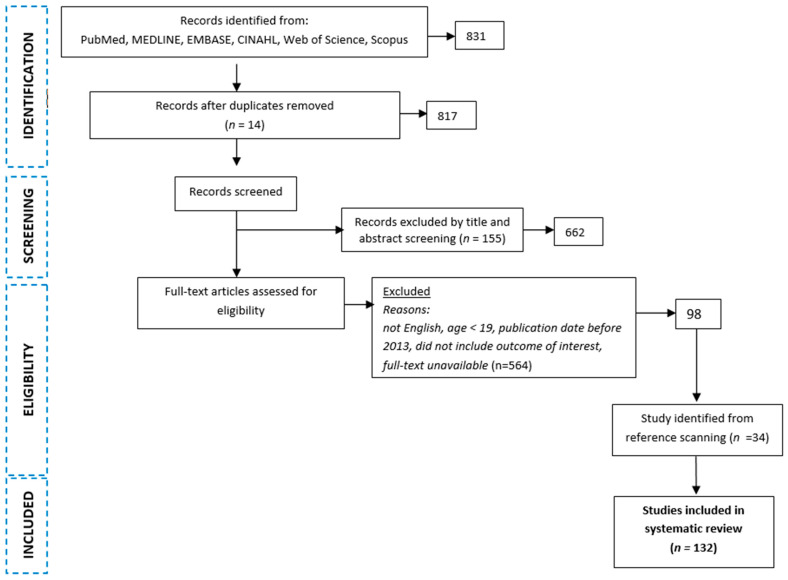
Flowchart of the analysis.

After searching databases and excluding articles that did not meet the criteria for inclusion in this systematic review, 98 full-text articles were obtained. A literature scan was then conducted and 32 additional articles were identified for inclusion. Ultimately, 132 full-text papers were included in this review (Figure 1).

## 3. Results

### 3.1. Standard Endpoint Definitions for TAVI

Complications after TAVI can be divided into procedural (short-term) and postoperative (long-term follow-up) (Table 1). Procedural complications mainly involve vascular issues, bleeding, arrhythmias, conduction disorders, coronary artery occlusion, myocardial infarction, stroke, perforation of the ventricular wall, valvular complications, dissection of aorta, aortic rupture, and death. Among distant complications occurring in the first few days after TAVI are vascular complications including bleeding, acute kidney injury, high-grade atrioventricular block, myocardial injury, cardiac tamponade, valve migration/embolization, valve dysfunction, and death.

The Valve Academic Research Consortium (the VARC-2) has standardized the definition of endpoints in studies evaluating the use of TAVI [55]. The risk of mortality, stroke, myocardial infarction, hemorrhagic complications, acute kidney injury, vascular complications, cardiac conduction disorders, and arrhythmias, as well as other relevant TAVI-related complications previously unclassified, must be assessed before a patient is eligible for treatment. As a secondary endpoint, VARC-2 identifies patients’ quality of life [8,56,57].

After mortality, the quality of life is the most frequently assessed endpoint of cardiac patients. TAVI provides an improved quality of life and is effective in alleviating AS symptoms [8,56,57]. TAVI is associated with positive changes in function in elderly patients at long-term follow-up [58]. In addition, performing TAVI in patients with severe AS prolongs life, reduces rehospitalizations for heart failure, and lowers the rate of death from cardiovascular causes to about 30% [59].

### 3.2. Determinants Affecting the Outcomes of Patients with Aortic Stenosis

**Figure 2:**
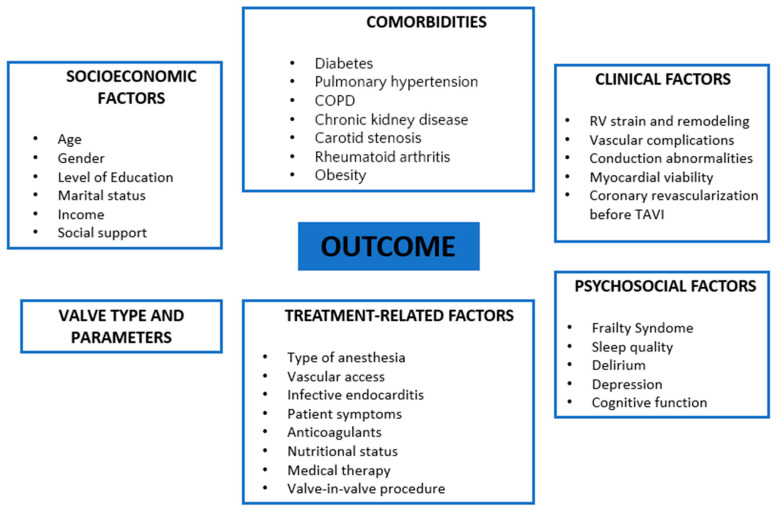
Determinants affecting the outcomes of patients with aortic stenosis undergoing TAVI.

Available studies have shown the association of dozens of factors related to the outcomes of AS patients undergoing TAVI, which can be divided into six main groups: psychosocial factors [21,28,39,40,41,42,43,44,45,58,60,61,62,63,64,65,66,67,68,69,70,71,72,73,74,75,76,77,78,79,80,81]; socioeconomic factors [9,10,33,34,35,36,37,38,82,83,84]; valve parameters [31,32,85,86,87,88,89,90]; comorbidities [26,47,48,49,50,51,52,53,54,87,91,92,93,94,95,96,97,98,99,100,101,102,103,104]; clinical factors [46,105,106,107,108,109,110,111,112,113,114,115,116,117,118]; and treatment-related factors [4,6,7,8,9,10,11,12,13,14,15,16,17,18,19,20,21,22,23,24,25,26,27,28] (Figure 2).

## 4. Factors Related to the Treatment

### 4.1. Anesthesia

Available studies do not support an association between the type of anesthesia and the incidence of mortality, major adverse cardiac and cerebrovascular events, infection requiring antibiotic treatment, and acute kidney injury 30 days after TAVI [4,6,7]. The authors present an inconclusive position on the most favorable type of anesthesia during TAVI. In the INSERT study [80] comparing the outcomes of patients after TAVI performed under general anesthesia (TAVI-GA) with sedation (TAVI-S), no differences were observed in surgical risk as determined by the EuroSCORE and STS score. In the case of quality of life, a study by Stańska et al. [8] suggests that patients’ quality of life increases after TAVI regardless of the type of anesthesia (general vs. local). The study by Neumann et al. [9] also found no significant differences between groups that received general anesthesia and sedation during TAVI.

Goren et al. [11] recommend that with the experience of the team, TAVI should be performed under local anesthesia due to the lack of safety differences between general and local anesthesia. This thesis is supported by available studies in which local anesthesia was significantly associated with a shorter procedure time and shorter hospital stay after the procedure [4,7]. More recent studies show that local anesthesia enables more effective detection of aortic regurgitation after TAVI [12]. General anesthesia (GA) may in turn be associated with significantly fewer paravalvular leaks (PVL) compared to sedation, which is related to the placement of a transesophageal echocardiography (TEE) probe during general anesthesia [13]. The opposite conclusion was reached by Zaouter et al. [14] who evaluated the incidence of PVL of moderate-to-severe severity 30 days after aortic valve implantation with TEE. Performing TAVI under GA with TEE was not associated with a lower incidence of moderate and severe PVL [14].

### 4.2. Vascular Access

The most common access for the TAVI procedure is through the common femoral artery (TF), either right or left, depending on the assessment of the diameter of the femoral and iliac arteries, the extent of atherosclerosis and calcification of their walls, and the tortuosity of the course of these vessels [15]. In 15–20% of patients who qualify for TAVI, femoral access is ruled out due to advanced atherosclerotic lesions [16]. In this group, valve implantation is performed via the subclavian/axillary artery (TSc), carotid arteries (TC), septal vena cava, direct aorta, or the apex of the heart (TA). Ferrari et al. [17] compared the clinical outcomes of 180 patients undergoing transapical (TA; n = 90) and transfemoral (TF; n = 90) procedures. The results of the study showed that the TA and TF groups had different risk profiles but mortality and adverse neurological outcomes occurred at similar rates. The TA approach is associated with more vascular complications and paravalvular leaks than TF [18]. Moreover, TF-TAVI is associated with fewer problems with usual activities and pain or discomfort and a higher quality of life compared with the TA-TAVI group [19]. In the study by Hudziak et al. [20], significantly more cases of pneumonia and blood transfusions were observed in the TA-TAVI group compared with the TC-TAVI group. The 30-day mortality, cardiac arrhythmia, and PPI rates were similar in the TC and TA groups. In the study by Al-Balah et al. [21], the procedure time appeared to be longer in the TSc group, while serious vascular complications were significantly more frequent in the TF group. Unfortunately, the literature does not have studies comparing all of the aforementioned vascular access types in terms of patient outcomes.

### 4.3. Infective Endocarditis

Despite the high incidence of comorbidities, patients undergoing TAVI have few infectious complications [119]. Compared to patients undergoing SAVR, patients undergoing TAVI have a significantly lower incidence of early (11.7% vs. 26.4%), intermediate (5.9% vs. 17.6%), and late (7.8% vs. 11.7%) postoperative infections [119]. The most commonly observed infectious complication after TAVI is infective endocarditis [120]. In a study by Bjursten et al. [121], the risk of prosthetic valve endocarditis (PVE) was 1.4% in the first year and 0.8% per year in subsequent years after TAVI. One-year survival after diagnosis of PVE was 58% and five-year survival was 29%. Staphylococcus aureus was more common in early (<1 year) PVE [121]. Studies have shown that infective endocarditis can lead to late PVE after TAVI with subsequent prosthesis regurgitation or obstruction [122,123].

### 4.4. Anticoagulants

The optimal anticoagulant treatment in the initial period after surgical implantation of an aortic valve bioprosthesis is becoming a subject of controversy due to the lack of high-quality scientific data. Many patients undergoing TAVI have comorbidities that require anticoagulant treatment. A recently published meta-analysis comparing the clinical outcomes and safety of novel anticoagulants (NOACs) and vitamin K antagonists (VKAs) in patients after TAVI showed a significantly higher reduction in thromboembolic risk in patients treated with VKAs compared to NOACs [22]. Recent guidelines do not mandate routine anticoagulant treatment after TAVI unless there are other indications for this treatment (e.g., comorbidities) [23]. An RCT by Rafiq et al. found that the use of VKAs for three months was associated with a significant increase in the incidence of major bleeding compared to ASA, while it did not lead to a reduction in the incidence of death or thromboembolic incidents. Therefore, the guidelines recommend considering ASA or VKA use for three months in all patients after surgical aortic valve bioprosthesis implantation [23]. Patients after TAVI without indications for chronic anticoagulant therapy require lifelong treatment with a first antiplatelet drug, such as ASA or clopidogrel. Dual antiplatelet therapy (DAPT) has been shown to be associated with more major or life-threatening bleeding, with no difference in the rate of ischemic incidents [24,25].

### 4.5. Symptoms of Patients

The presence of symptoms indicative of severe aortic stenosis is one of the eligibility criteria for treatment of the defect. Available studies confirm that the long-term outcomes of patients with NYHA class I are better than those with NYHA class IV or III [124]. In a study by Adamo et al., the presence of NYHA class IV in TAVI candidates was associated with a significantly increased risk of death at three months; after three months, the risk of death was comparable to patients with other NYHA classes [125].

In a retrospective study by Kvaslerud et al. [126], asymptomatic patients with severe aortic valve stenosis who were advised against surgery had a significantly higher mortality rate than patients who had their aortic valve replaced. In contrast, a recent RCT showed that for asymptomatic patients, an early TAVI strategy was superior to clinical surveillance in reducing the incidence of death, stroke, or unplanned hospitalization for cardiovascular causes [127].

### 4.6. Medical Therapy

Cardiac remodeling is crucial to the prognosis of patients with AS and may affect the outcomes resulting from aortic valve replacement. Nearly half of patients are hospitalized due to heart failure one year after the TAVI procedure [128]. Recent studies show the benefits of sodium-glucose cotransporter-2 (SGLT2i) inhibitors in improving cardiac remodeling and long-term outcomes in patients undergoing TAVI. In the study by Scisciola et al., in patients with LF-LG AS, the SGLT2 gene and protein were overexpressed in cardiomyocytes and were associated with myocardial fibrosis, inflammation, and oxidative stress [129]. SGLT2i administration to diabetic patients with severe AS, LVEF < 50%, and EVCD undergoing TAVI was associated with more favorable cardiac remodeling and a reduced risk of MACE at two-year follow-up [130].

### 4.7. Nutritional Status

The results from some studies suggest an association of nutritional status with clinical outcomes in surgically treated patients with ankylosing spondylitis [26,27,28]. In a study by Taniguchi et al. [27], low appetite status just before discharge of patients undergoing TF TAVI was significantly associated with more frequent major adverse cardiovascular and cerebrovascular events (MACCE). In addition, the inadequate nutritional status of patients undergoing the procedure increases the risk of infection and decreases the likelihood of recovery.

### 4.8. Valve-in-Valve Procedure

A comparison of ViV-TAVI (bioprosthetic valve implantation) and NV-TAVI (native valve implantation) shows comparable mortality and stroke rates, confirming previous studies [131,132,133]. ViV-TAVI causes less strain on the heart muscle, resulting in a lower risk of permanent pacemaker implantation [134]; however, the procedure carries the risk of coronary obstruction, which can be assessed by CT measurements [135].

ViV-TAVI is associated with higher rates of readmissions, long-term mortality, higher post-procedure gradients, and greater paravalvular leaks, which may reduce long-term efficacy [136]. Data on the durability of ViV-TAVI are still limited, especially for small bioprostheses [133].

A study comparing SE and BE valves in ViV-TAVI suggests that SE valves may have lower complication rates and better hemodynamic outcomes, especially with smaller valves [137]. Higher bleeding rates for BE valves may be due to larger sheath sizes [31]. Current guidelines do not favor any type of valve [1].

## 5. Socioeconomic Factors

As far as the literature is concerned, the evidence on the impact of sociodemographic factors on patient outcomes after TAVI is limited. Studies have shown better outcomes among married patients who underwent a cardiovascular procedure, including TAVI [9]. In Newell et al.’s study [82], married women (*p* = 0.041) had lower one-year survival rates than single women. On the contrary, married men (*p* = 0.007) had a higher survival rate than their single counterparts. In addition, widowed patients had a higher 30-day readmission rate.

A Swedish study emphasized the need to provide social support to patients 80 (7.4) years old who were offered TAVI [138]. Patients specifically noted difficulties in daily social functioning, coping with disease symptoms, and adjusting to altered health conditions [33]. Mohee et al. [34] attempted to clarify whether socioeconomic status affects patient outcomes after TAVI. Patients with lower socioeconomic status were at risk of longer hospital stays compared with patients with higher status. In contrast, 30-day, one-year, and three-year survival/mortality rates were similar in all patients regardless of socioeconomic status; however, the association of education level with perioperative and mid-term outcomes was not confirmed [10].

### Age and Gender

Researchers present conflicting opinions on the effect of age on the outcomes of patients after TAVI valve implantation. In Eichler et al.’s study [35], higher mortality was significantly associated with older patient age; however, multivariate analysis also confirmed the association of a number of comorbidities, diabetes, low left ventricular ejection fraction (LVEF), a higher EuroSCORE score, a low score in the nutrition domain, and for mobility in the frailty index questionnaire. In Olasinska-Wisniewska et al.’s [36] study, age was not a factor associated with the TAVI outcomes. The incidence of postoperative complications was similar in the ≥85 years group and in the <85 years group. Major, life-threatening, and minor hemorrhagic complications, as well as vascular access site complications, did not differ between the two groups, and the incidence of myocardial infarction and stroke was comparably low in both groups. In-hospital mortality and one-year mortality did not differ between the groups. Thus, it can be concluded that TAVI in patients 85 years of age and older is still a relatively safe procedure, and age alone should not be a discriminating factor from qualifying for TAVI. The opposite conclusion was reached by Delijani et al. [37], who proved that patients aged 80–89 years and older than 90 years undergoing TAVI have an increased risk of rehospitalization, complications, and mortality compared with patients younger than 70 years. In the Polish study by Dabrowski et al. [38], the EuroSCORE II-defined perioperative risk and one-year cardiovascular mortality were significantly higher in older patients undergoing TAVI. The rate of complications after TAVI was also dependent on patient age. Patients differed significantly in the incidence of vascular and hemorrhagic complications, which were more common in older patients.

In addition to patient age, the literature has evidence of an association of gender, socioeconomic status, and marital status with the outcomes of patients with AS after TAVI. In a study by Shah et al. [84], the overall in-hospital mortality rate among female patients undergoing TAVI was higher compared with men; moreover, female gender was an independent predictor of referral to a rehabilitation center after TAVI.

## 6. Psychological Factors

There are scarcely any studies on the impact of psychosocial factors on the outcomes of patients undergoing TAVI.

### 6.1. Frailty Syndrome

Recently published guidelines from the European Society of Cardiology (the ESC) [5] emphasize the importance of a pre-interventional assessment of frailty syndrome in patients scheduled for cardiac surgery or TAVI. According to Bertschi et al. [60], the frailty index—but not the cardiac risk scores (STS and EuroSCORE)—identifies patients at increased risk of functional deterioration (ADL) after TAVI. Identifying patients with a high frailty index prior to TAVI is clinically important because these patients may benefit from targeted geriatric treatment and rehabilitation after TAVI [5]. Although the probability of a poor outcome is high, patients with frailty syndrome also have a high probability of a favorable long-term functional outcome through targeted strategies to optimize pre-intervention management (e.g., pre-rehabilitation) [5].

Comprehensive geriatric assessment helps to prevent TAVI complications and provides a rapid assessment of perioperative and postoperative complications that occur [61,62]. Frailty syndrome is associated with worsening quality of life (QoL) one year after TAVI. Moreover, QoL improved in frail patients without peripheral artery disease or renal impairment at the start of the study. In Skaar et al.’s study [39], patients with higher GA frailty scores had significantly higher two-year mortality after TAVI. The modified Essential Frailty Toolset (EFT) using four parameters of frailty—gait speed, the Mini Mental State Examination (MMSE), anemia, and hypoalbuminemia—was an independent predictor of late bleeding after TAVI [95].

### 6.2. Postoperative Non-Alcoholic Delirium

Postoperative delirium (PD) is another factor that may be related to functional outcomes and quality of life in patients after TAVI [63,64]. PD significantly increased the likelihood of death in the first year after the procedure. PD occurs more frequently in patients aged ≥80 years with severe aortic stenosis after SAVR compared to TAVI [65] and in TA (47%) compared to TF TAVI (17%) [66]. In a prospective study by Beishuizen et al. [40], delirium resulted in a fourfold increased likelihood of functional deterioration or death in the first year after the procedure. Delirium after TAVI predicted greater Activities of Daily Living (ADL) and Instrumental Activities of Daily Living (IADL) disability after one month but not after six months of follow-up [67]. Individuals who developed delirium after SAVR and TAVI had worse short-term IADL function but did not appear to have long-term impairment in physical health, mental health, or self-esteem. PD in patients aged >80 years after aortic valve treatment may be a major risk factor for postoperative morbidity and mortality [67].

### 6.3. Cognitive Functions

Cognitive deficits are a predictor of PD and mortality after TAVI [28,40,68,69]. Cognitive impairment is a very common geriatric syndrome in elderly patients with severe aortic stenosis that is associated with functional disability in daily activities [70]. Cognitive impairment is associated with postoperative adverse events in elderly patients undergoing TAVI according to the Clavien–Dindo classification, which is used to assess the severity of surgical complications and based on the type of therapy needed to correct the complication [71]. In high-risk patients, both TAVI and SAVR are associated with significant improvements in quality of life up to one year without detrimental effects on cognitive function [72].

A meta-analysis by Khan et al. [41] observed no significant changes in cognitive function in patients after TAVI, suggesting that cognitive performance after TAVI is preserved. Moreover, TAVI may improve cognitive function [58,139] associated with increased cerebral perfusion [73] in older patients with severe AS [42,44]. In the CLEAN-TAVI randomized clinical trial among patients with severe aortic stenosis undergoing TAVI, the use of a brain-protective device using the Claret Montage Dual Filter System (Claret Medical Inc., Santa Rosa, CA, USA) reduced the incidence of ischemic cerebral lesions in potentially protected areas [43]. Early deterioration of some complex cognitive functions was observed in a quarter of TAVI recipients and persisted at one year in 10% of patients [44]. In a study by Monnin et al. [140], the clinical course of patients with mild-to-moderate cognitive decline did not differ at one year after TAVI compared with patients without cognitive impairment.

### 6.4. Sleep Disturbance

One of the symptoms accompanying patients with AS is sleep disturbance [74]. In the immediate postoperative phase, sleep difficulties are reported by both surgically treated patients and patients undergoing TAVI [75,76,77]. Lorenzoni et al. [75] showed a small significant improvement in sleep quality, as well as small positive changes in self-care and usual activities; however, no correlation was detected between quality of life and functional status and sleep quality.

### 6.5. Anxiety and Depression

Depression reduces the quality of life of patients with cardiovascular disease and limits the achievement of benefits from modern treatment [78,79]. Depression in patients after TAVI is associated with high mortality five years after the procedure [80]. A decrease in the incidence rate of depression was also observed among patients who underwent TAVI, which may be related to the resolution or alleviation of AS symptoms after the intervention [45,78]. In addition, higher overall quality of life correlated with lower levels of depression in patients after TAVI. On the other hand, a study by Bäz et al. [81] proved the prognostic significance of anxiety and depression but only at lower stages of aortic stenosis.

## 7. Clinical Parameters

### 7.1. RV Strain and Remodeling

The decision to perform TAVI is not based on structural and functional assessment of the right ventricle. According to recent studies, both structural and functional alterations of the right heart predicted worse clinical outcomes in patients undergoing TAVI [105,106]. Moreover, right ventricular (RV) dysfunction reflects advanced left ventricular (LV) dysfunction [107]. The assessment of tricuspid annulus shape in the end-diastole (ED) may be a useful parameter for risk stratification in patients undergoing TAVI [108]. Circular tricuspid annulus shape remodeling in the ED is associated with greater right atrial and ventricular dilatation and higher long-term mortality after TAVI [108]. Studies have shown the limited usefulness of left ventricular dysfunction in predicting distant complications after TAVI [117]. In a study by Asami et al. [46], RVD at baseline was associated with a more than twofold increased risk of cardiovascular death at one year after TAVI. Only a few studies not considering right ventricular parameters in mortality risk assessment have confirmed the predictive properties of left ventricular function in stratifying the risk of distant mortality [109,110].

Tricuspid regurgitation (TR) is also not infrequently in conjunction with AS [111,112]. Concurrent significant TR is associated with worse outcomes but its occurrence should not change the approach to AS treatment (i.e., from TAVI to surgical or conservative).

### 7.2. Advanced Imaging Parameters—Cardiovascular Magnetic Resonance Imaging

Cardiovascular magnetic resonance imaging (CMRI) provides a comprehensive evaluation of the aorta and peripheral vessels. It identifies atherosclerotic plaques, amyloid, and complex anatomy such as aneurysm, dissection, intramural hematoma, and congenital defects [141]. It can provide essential information in making decisions about access sites, ring dimensions, ostium height, LVOT dimensions, and predicting complications [141].

CMRI allows for the assessment of prosthetic valve stability and function, which is crucial for clinical outcomes after TAVI. It is particularly useful in assessing bicuspid regurgitation, thrombus, and left ventricular remodeling after TAVI [141]. CMRI can detect thrombus but its temporal and spatial resolution may be insufficient for small thrombi [142]. However, an increase in bioprosthetic valve gradient may indicate their presence [141].

Paravalvular leak (PAR) remains a significant challenge after TAVI, affecting short- and long-term mortality, even in mild cases [143]. Accurate assessment of PAR is of great prognostic importance. CMRI, using a velocity mapping (PC) technique, is proving more accurate than echocardiography in assessing PAR, especially for balloon-expandable and self-expandable implants [144,145]. CMRI often ranks PAR severity higher than echocardiography, highlighting its diagnostic advantage [141].

Assessment of myocardial viability based on late gadolinium enhancement (LGE) is now fundamental to understanding the prognosis of patients after TAVI [146]. Coronary revascularization before TAVI reduces the relative risk of mortality from any cause, MI, or urgent revascularization [147]. In contrast, documentation of an ischemic LGE pattern is associated with a worse outcome after revascularization and a higher risk of MACE [148]. In patients with structural and functional cardiac remodeling, the absence of transmyocardial LGE is a favorable prognostic marker for the return of left ventricular dysfunction after flow restoration [149].

### 7.3. Vascular Complications

Malyar et al. [113] noted the incidence of critical limb ischemia (CLI) and its impact on in-hospital outcomes in patients undergoing TAVI for severe AS. The overall in-hospital mortality among patients undergoing TAVI without peripheral artery disease (PAD), PAD without CLI, and CLI was 6.1%, 8.4%, and 14.7%, respectively (*p* < 0.001). This means that in patients undergoing TAVI, the presence of PAD is associated with an increased risk of perioperative complications, while only CLI independently predicts increased in-hospital mortality.

### 7.4. Conduction Abnormalities (Right/Left Bundle Branch Blocks and Atrioventricular Block)

The rate of permanent pacemaker implantation after TAVI varies between 3.4% and 25.9% [150].

Pre-procedural right bundle branch block (RBBB) is common in patients with severe AS undergoing TAVI and is associated with significantly higher rates of pacemaker implantation (PPM) but not with worse short- and mid-term outcomes. There is currently no evidence of prophylactic pacemaker implantation before TAVI in patients who do not meet the standard indications for pacemaker implantation (including RBBB).

Post-procedural new conduction abnormalities: complete or high-degree atrioventricular block (AVB) persists for 24–48 h after TAVI or appears later, and new-onset alternating bundle branch block requires a permanent pacemaker [150].

Regarding pre-existing RBBB, new transient high-degree AVB, prolongation of PR, or an axis change requires a permanent pacemaker [150].

Due to the significant anatomical proximity of the aortic valve and the left bundle branch, the most common conduction disturbance after TAVI is a new-onset LBBB [114]. In the case of persistent new LBBB, QRS > 150 ms or PR > 240 ms requires ambulatory electrocardiogram (ECG) monitoring or electrophysiology study (EPS) but not a mandatory permanent pacemaker. Several reports suggest that new-onset LBBB after TAVI is associated with decreased LV function and long-term mortality hazard [115,116,118].

### 7.5. Valve Type and Parameters

The available studies indicate that the type and route of the implanted valve also affect the course of the procedure and the therapeutic outcome of the patient after TAVI. Researchers dispute which valves are associated with a lower risk of complications during the procedure and distant complications. Attention is paid to different outcomes depending on the design of the valve. Two basic designs of the valve are available nowadays—a self-expanding valve (SEV) and a balloon-expandable valve (BEV). We cannot compare them in every aspect, because, for example, only the BEV can be implanted via a transapical approach.

In the CHOICE trial, among patients with high-risk aortic stenosis undergoing TAVR, the use of a balloon-expandable valve resulted in a higher device success rate than the use of a self-expandable valve (lower rate of residual aortic regurgitation, less-frequent need to implant more than one valve, lower mortality, and lower rate of pacemaker implantation) [151]. Measurable parameters may influence outcomes. The BEV shows an advantage in studies when the following factors are taken into account: the occurrence of atrioventricular block and need for permanent pacemaker implantation (PPI), PVL, and the incidence of stroke [32,85,86,87,88]. In bicuspid anatomy, the BEV has a better outcome [31,89]. Due to implantation technique renal complications, acute kidney injury was more common with the SEV due to using additional contrast for repositioning [87]. However, the SEV is characterized by a smaller gradient assessed in the TTE after implantation, and for this reason, some authors postulate that it should be preferred in patients with a small aortic annulus [85]. If access to the coronary arteries is needed in the future after TAVR, then the BEV is the safer option. Due to the design of the valve, i.e., supra-annular in the case of the SEV and intra-annular for BEV, the short frame of the BEV allows direct access to the coronary artery, unlike the SEV, where catheterization is performed through the frame, which does not always allow access [90].

## 8. Usefulness of Regression Models of Scales Considering Multi-Morbidity in Predicting Perioperative and Distant Complications After TAVI

The coexistence of additional chronic diseases in patients undergoing TAVI is key to the course of treatment and outcome. Available studies confirm significant differences in the near and distant prognosis of patients with diabetes [47,91], pulmonary hypertension [92,93], chronic obstructive pulmonary disease (COPD) [48,49,50,94,95], chronic kidney disease [51,52,96,97], carotid stenosis [53,54,98,99,100], rheumatoid arthritis [26,87], and obesity [101,102,103,104] compared to patients without these comorbidities. Given the prevalence of multi-morbidity in patients referred for TAVI and the wide range of therapeutic options at each stage of preparation for TAVI and after TAVI, the lack of a dedicated tool to assess the risk of perioperative and distant complications after TAVI is noteworthy. General surgical risk assessments remain important to objectively support the identification of patients unsuitable for SAVR but there is a lack of risk models after TAVI [1].

Among the scales that address the most common comorbidities and possible conditions associated with vascular intervention are the Charlson Comorbidity Index (the CCI), MELD, C2HEST risk score, HAS-BLED, CHA2DS2-VASc, and CHILD-PUGH. Previous studies show the widespread use of these simple tools for predicting near and distant risk in patients with diseases for which dedicated, specific prognostic tools have not been developed [152,153,154,155]. The use of scales allows for a broad assessment of seemingly irrelevant prognostic factors. In addition, these scales are used to partially assess factors whose predictive value has already been confirmed, such as frailty syndrome (CCI) or malnutrition (MELD, CHILD-PUGH). Therefore, given the above, it is important to pay attention to evaluating the usefulness of scales for multi-morbidity in TAVI patients.

## 9. Study Limitations and Practical Implications

This systematic review has several limitations. Despite the large number of included studies, it is not possible to clearly identify which variables have a significant impact on patient prognosis due to the use of different vascular access types for prosthesis implantation. Some of the cited studies refer to older-generation prostheses that are not currently used by implanters. Comparing older-generation prostheses with newer ones can be misleading. Another limitation of this review is the reference to the VARC-2 criteria, instead of the newer VARC-3, due to the criterion for selecting articles until 2023. It should be noted that the cited studies only described the effect of one or more variables on patient outcomes after TAVI at different time points after the procedure. Another limitation of this study is the lack of quantitative meta-analysis of the data. The paper focuses on a qualitative synthesis of available studies, which limits the ability to present the statistical impact of individual prognostic parameters. Therefore, in the future, a multivariate analysis of variables should be performed to create a predictive model to stratify the perioperative risk of patients who qualify for TAVI.

## 10. Conclusions

This systematic review showed that numerous factors that can affect the outcomes of patients undergoing aortic valve replacement depend on the method of treatment, the type of valve, patients’ compliance, and the individual characteristics of the patient in question (Table 2). In the studies reviewed, due to the paucity of evidence on patient-related factors, the focus was on the role of the treatment method in achieving specific endpoints for patients with AS (mortality, QoL, and incidence of early and late complications related to TAVI). Most studies have emphasized the importance of technique, valve placement, and valve type. There are conflicting data on the impact of paravalvular leak and left ventricular dysfunction on the outcomes of AS patients treated with TAVI. Although the number of known factors influencing the outcomes of TAVI patients is increasing, more research is needed on new factors limiting patients’ achievement of therapy benefits. Due to the divergent views of researchers on some of the factors influencing patient outcomes after TAVI, further analysis is needed to develop a new tool for assessing predictive outcomes in this group of patients and to provide valuable scientific evidence for this currently unmet clinical need.

## Figures and Tables

**Table 1:** TAVI-related complications.

Author, [Ref]	Peri-Procedural Complications and 30-Day All-Cause Mortality After TAVI														
	Bleeding	AF	LBBB/RBBB	AVB/Pacemaker Implantation	Coronary Artery Occlusion	MI	Stroke	Vascular Complications	AKI	Myocardial Injury	Infections	Cardiac Tamponade	Valve Migration/Embolization	Valve Dysfunction	30-Day All-Cause Mortality
Van Belle E., 2020 [31]	8.1–10.2%					0.1–0.4%	1.8–2.5%	6.4–7.5%							3.8–5.6%
Vlastra W., 2019 [32]	5.5–6.6%	5.5–5.6%				0.6–0.7	1.5–2.6%								5.3–6.2%
Thiele H., 2020 [6]			6.39–6.88%	0.46–14.7%					8.84–9.39%						
Neumann F.-J., 2020 [9]	4.1–4.7%	4.7–6.8%				0.1–0.2%	0.6–1.7%	4.2–4.4%	0.4–1.5%						1.7–2.1%
Goren O., 2015 [11]	3%	37%					2%	3%	2%						
Romano S.M., 2020 [12]		13–26%		16%			2–3%	3–22%	28–46%						
Oguri A., 2014 [13]	1.6–8.1%					1.6%	0.7–2.1%	5.6–6.1%	1.5%			2.1%			
Ferrari E., 2017 [17]	4%						3%	7%	2%				2%		
Taniguchi Y., 2019 [27]							1.4–4.8%								
Deharo P., 2020 [28]							2.88–3.68%								
Shigetomi K., 2023 [33]							18.8%								
Mohee K., 2019 [34]	0					0.9%	0.3%	1.5%				0.9%	0.7%		
Chabová B., 2022 [10]	2–3%					0–1%	2%	4%							3–4%
Eichler S., 2018 [35]	4.4%						2%	8.1%	2.6%		2%				
Olasinska-Wisniewska A., 2017 [36]	7.4–10.3%					0.7%	0.7%	7.4–9.6%	38.2%						4.4–7.7%
Delijani D., 2023 [37]						2.5%	1.8%	1.3%	10.5%						
Dąbrowski M., 2021 [38]	4–18.5%				0.7–1%	0.7–1%	1.2–3%	3.1–10%							4.3–5.5%
Skaar E., 2019 [39]	19.7%				0.7%		2.1–2.8%	4.2%	2.1%						2.8%
Beishuizen S.J., 2020 [40]	28.6–31.2%					0	2.6%	14.3–15.6%	5.2–7.1%						7.8–28.6%
Khan M.M., 2019 [41]							3.4–6%								3.4–7.7%
Tsuchiya S., 2020 [42]	7%	7%				0	0	7%	0						
Haussig S., 2016 [43]	2–10%	14%		16%				4–12%							
Auffret V., 2016 [44]	9.8%	9.8%				2%	0	3.9%	2%						
Tamm A.R., 2023 [45]		9.5%		19%			1.7%	12.5%							3.6%
Asami M., 2019 [46]	7.6–16.3%					0.8–1.9%	2.5–3.2%	9.8–11.1%	2.4–6.6%						2.7–9.9%
Mendez-Bailon M., 2017 [47]											1.82–2.45%				
Mach M., 2019 [48]	3.8–5.2%			10.4–10.5%		0.2–0.4%	0.4–0.8%	3.7–4.3%	5.3–6.5%		4.2–7.4%				4.8–5.3%
Xiao F., 2020 [49]	43.3%								20.7%		2.7%				
de Miguel-Diez J., 2020 [50]				13.98%							1.17%				
Jäckel M., 2023 [51]				22.7%											
Witberg G., 2021 [52]									2.8–15.6%						
Lepidi S., 2022 [53]							2.8%								
Oestreicher S., 2023 [54]	1.8–2.6%	4.1–5.3%		14.8–16.1%		0–1%	2.3–7.1%	3.6–4.6%					0–2%		1.8–2%

AF: atrial fibrillation; LBBB: left bundle branch block; RBBB: right bundle branch block; AVB: atrioventricular block; MI: myocardial infarction; AKI: acute kidney injury.

**Table 2:** Factors determining peri-procedural and long-term outcomes of patients after TAVI.

Outcome		Factors Decreasing the Occurrence of the Outcome	Factors Increasing the Occurrence of the Outcome
Peri-procedural	Stroke	Early TAVI of patients with severe AS without symptoms; use of Claret Montage Dual Filter System (Claret Medical Inc.); balloon-expandable valve	
	Pacemaker implantation	Valve-in-valve; balloon-expandable valve	Pre-procedural right bundle branch block
	Moderate paravalvular leaks	Balloon-expandable valve	Transapical access; valve-in-valve; sedation
	Bleeding		Balloon-expandable valve
	AKI	Sedation	Self-expanding valve
	Vascular complications		Transapical access; age > 80
	Coronary artery occlusion		Valve-in-valve
	Myocardial infarction	Sedation, Coronary revascularization before TAVI	
	Infections		Transapical access; malnutrition
	Valve migration/embolization	Balloon-expandable valve	
	In-hospital mortality		Women; critical limb ischemia;
	Length of hospital stay	Sedation	Lower socioeconomic status; critical limb ischemia; peripheral artery disease
Long-term	Mortality (3 month)	Early TAVI of patients with severe AS without symptoms	NYHA 3 and 4; no symptoms of severe AS; valve-in-valve
	Mortality (≥12 month)	Early TAVI of patients with severe AS without symptoms; married men; balloon-expandable valve; Coronary revascularization before TAVI	NYHA 3 and 4; no symptoms of severe AS; valve-in-valve; age > 80; married women; frailty; postoperative delirium; cognitive impairment; depression; circular remodeling of tricuspid ring shape in end-diastole; right ventricular dysfunction; left ventricular dysfunction; paravalvular leak; new onset of left bundle branch block after TAVI; more comorbidities; lower left ventricular ejection fraction; diabetes; malnutrition; impaired functional status; higher EuroSCORE; tricuspid regurgitation
	30-day mortality		Sedation
	Valve dysfunction	Balloon-expandable valve	
	Bleeding		Slower walk; cognitive impairment; anemia; hypoalbuminemia
	Hospitalizations	Early TAVI of patients with severe AS without symptoms	Valve-in-valve; age > 80; single marital status
	Functional status	Postoperative delirium	
	High level of quality of life	Transapical access; frailty; depression	
	MACE	SGLT2i	Malnutrition; age > 80; an ischemic LGE pattern

AS: aortic stenosis; SGLT2i: sodium-glucose co-transporter-2 inhibitors; TAVI: transcatheter aortic valve implantation; NYHA: The New York Heart Association Functional Classification; AKI: acute kidney injury; LGE: late gadolinium enhancement.
